# Metabarcoding of shrimp stomach content: Harnessing a natural sampler for fish biodiversity monitoring

**DOI:** 10.1111/1755-0998.12956

**Published:** 2018-11-09

**Authors:** Andjin Siegenthaler, Owen S. Wangensteen, Ana Z. Soto, Chiara Benvenuto, Laura Corrigan, Stefano Mariani

**Affiliations:** ^1^ Ecosystems and Environment Research Centre, School of Environment and Life Sciences University of Salford Salford UK; ^2^ Environment Agency Tyneside House Newcastle upon Tyne UK; ^3^ Present address: Department of Integrative Biology University of Guelph Guelph Ontario Canada; ^4^ Present address: Norwegian College of Fishery Science, UiT the Arctic University of Norway Tromsø Norway

**Keywords:** biodiversity monitoring, *Crangon crangon*, DNA, environmental assessment, fish communities, trophic metabarcoding

## Abstract

Given their positioning and biological productivity, estuaries have long represented key providers of ecosystem services and consequently remain under remarkable pressure from numerous forms of anthropogenic impact. The monitoring of fish communities in space and time is one of the most widespread and established approaches to assess the ecological status of estuaries and other coastal habitats, but traditional fish surveys are invasive, costly, labour intensive and highly selective. Recently, the application of metabarcoding techniques, on either sediment or aqueous environmental DNA, has rapidly gained popularity. Here, we evaluate the application of a novel, high‐throughput DNA‐based monitoring tool to assess fish diversity, based on the analysis of the gut contents of a generalist predator/scavenger, the European brown shrimp, *Crangon crangon*. Sediment and shrimp samples were collected from eight European estuaries, and DNA metabarcoding (using both 12S and COI markers) was carried out to infer fish assemblage composition. We detected 32 teleost species (16 and 20, for 12S and COI, respectively). Twice as many species were recovered using metabarcoding than by traditional net surveys. By comparing and interweaving trophic, environmental DNA and traditional survey‐based techniques, we show that the DNA‐assisted gut content analysis of a ubiquitous, easily accessible, generalist species may serve as a powerful, rapid and cost‐effective tool for large‐scale, routine estuarine biodiversity monitoring.

## 1 INTRODUCTION

Accurate and reliable estimates of biodiversity and species distributions are essential for successful ecosystem management and environmental policy (Hooper et al., 2005; Rees, Maddison, Middleditch, Patmore, & Gough, 2014). Understanding biodiversity changes in coastal systems, such as estuaries, is of special interest since these provide essential ecosystem functions and services and are heavily affected by anthropogenic pressures (Halpern et al., 2008; Sheaves, Baker, Nagelkerken, & Connolly, 2015). Estuaries are highly productive systems, providing food and shelter for a large range of fish and invertebrates (Beck et al., 2001; Heip et al., 1995). These habitats act as important nurseries for many fish species, resulting in a greater density, survival rate and growth of juveniles than surrounding habitats (Beck et al., 2001; Kraus & Secor, 2005), which explains the adaptations and energy required for fish larvae to migrate from the open sea to estuaries (Huijbers et al., 2012; Norcross & Shaw, 1984). Alongside their importance as nursery areas, estuaries also support a wide range of adult fish species including estuarine residents, marine and freshwater “stragglers” (taxa normally occurring in marine habitats), and migratory species (Elliott & Dewailly, 1995; Elliott et al., 2007). Many of these are important targets for fisheries or key‐stone elements for coastal food webs and of relevance for global economy and food security (Jovanovic, Longmore, O'Leary, & Mariani, 2007; Pauly, Watson, & Alder, 2005; Scheffer, Carpenter, & Young, 2005; Wilson, 2002). An understanding of the community structure, spatial distribution, population connectivity and prey selection of bony fish is important for ecosystem characterization and management (Genner et al., 2004; Jovanovic et al., 2007; Kraus & Secor, 2005; Mariani, Boggan, & Balata, 2011). This is becoming crucial since ichthyofaunal communities are under pressure from a range of anthropogenic impacts such as overfishing, pollution and climate change (Courrat et al., 2009; Genner et al., 2004; Wilson, 2002). Due to these pressures, fish communities are generally considered to be suitable biological indicators for the environmental quality of estuarine systems, as monitoring fish communities integrates the direct and indirect effects of stressors on the entire aquatic ecosystem (Fausch, Lyons, Karr, & Angermeier, 1990; Whitfield, 2002). Fish surveys are regularly conducted for the management of oceanic and transitional waters, fisheries stock assessments, detection of invasive species, monitoring of environmental changes, water quality assessments, etc. (Pyšek & Richardson, 2010), and are required to comply with environmental policy such as the EU Water Framework Directive for Transitional Waters (Ferreira et al., 2007).

Traditional estimates of fish diversity largely depend on fish captures, which are usually invasive, costly, labour intensive and selective (Cotter et al., 2004; Lapointe, Corkum, & Mandrak, 2011; Thomsen et al., 2012). Recent molecular biodiversity assessment methods, such as environmental metabarcoding, focus on detecting animals' presence by collecting the DNA they have left behind in the environment (Taberlet, Coissac, Pompanon, Brochmann, & Willerslev, 2012; Thomsen et al., 2012) and applying high‐throughput sequencing (HTS) to identify multiple taxa based on bulk DNA extracted from a community (DNA derived from many individual organisms, representing several species) or environmental sample (i.e., water, soil, faeces; Barnes & Turner, 2016). Metabarcoding can successfully identify small, cryptic or decomposed specimens with reduced cost and effort compared to traditional methods, and is independent of the species' developmental stage (Chariton et al., 2015; Hajibabaei, Shokralla, Zhou, Singer, & Baird, 2011; Lejzerowicz et al., 2015; Leray & Knowlton, 2015), though this may also represent a limitation, when that type of information is required (Valentini et al., 2016).

A recent metabarcoding development is the use of DNA detected in the gut contents of parasitic/predatory organisms to estimate the diversity and distribution of their prey items. Molecular trophic tools have advantages over traditional taxonomic methods since the stomachs of animals often contain a high proportion of material that is very difficult to identify with traditional microscopic identification, such as small, soft bodied and highly digested prey (McClenaghan, Gibson, Shokralla, & Hajibabaei, 2015; Symondson, 2002). The application of leeches and carrion flies as biodiversity sampling tools has been proposed for the rapid assessment of mammals in several terrestrial habitats (Calvignac‐Spencer, Merkel, et al., 2013; Schnell et al., 2015, 2012). Although the concept of examining species distribution based on their detection as prey items in the stomach contents of predators has been applied using traditional morphological methods (e.g., Boucek & Rehage, 2014; Fahrig, Lilly, & Miller, 1993; Lasley‐Rasher, Brady, Smith, & Jumars, 2015; Stevens, Blewett, Champeau, & Stafford, 2010), trophic DNA‐based methods for biodiversity assessment have not yet been employed in marine systems (though suggested by Boyer, Cruickshank, & Wratten, 2015; Deiner et al., 2017), and much still needs to be done in order to identify the most appropriate sample types and markers to detect specific biodiversity components, such as, for instance, teleost species (Shaw et al., 2016).

Here, we focus on the applicability of metabarcoding of DNA extracted from the stomach contents of an opportunistic scavenger/predator, the brown shrimp, *Crangon crangon* L., as a sampling tool for fish diversity in European coastal waters. The brown shrimp is a widespread and abundant decapod crustacean and vital component of the benthic food web in European soft bottom habitats (Bamber & Henderson, 1994; Campos & van der Veer, 2008), and represent an important target for fisheries, with catches up to 35,000 tons in 2011 (Aviat, Diamantis, Neudecker, Berkenhagen, & Müller, 2011; Campos & van der Veer, 2008). Showing a highly opportunistic diet, the brown shrimp consumes a wide variety of food items, ranging from meiofauna to fish (Evans, 1983; Oh, Richard, & Richard, 2001). Though being a generalist, it prefers larger motile prey items, including commercially important teleost species (Siegenthaler, Wangensteen, Benvenuto, Campos, & Mariani, 2018; van der Veer & Bergman, 1987). Its opportunistic diet, in combination with its high abundance (van der Veer, Feller, Weber, & Witte, 1998) and ease of catch (e.g., by push‐net or bycatch; Gamito & Cabral, 2003), makes the brown shrimp a very suitable candidate as an estuarine biodiversity sampling tool. Besides the main general objective, to evaluate the suitability of shrimp stomach content to assess fish diversity, two secondary objectives were addressed, namely i) to compare the efficacy of different DNA sample media to detect fish taxa and ii) to compare fish communities identified via metabarcoding surveys with those identified from concurrent net surveys.

## 2 METHODS

This study comprised two independent and complementary efforts. First, DNA extracts from *C. crangon* stomach and sediment samples collected from six European estuaries were amplified with two different markers (COI and 12S) and the detected fish taxa were evaluated and compared in relation to sample type, marker and location. Sample collection and COI amplification was conducted as part of a larger study focusing on the use of metabarcoding to assess the trophic ecology of *C. crangon* (Siegenthaler et al., 2018). No water samples were collected, and no ichthyofaunal surveys were conducted during this study. Therefore, a second complementary, but smaller, study was conducted focussing on two British estuaries to compare the detection of fish environmental DNA (eDNA) extracted from shrimp stomachs, water and sediment substrates, with morphological identification of fish caught in concurrent seine net surveys.

### 2.1 Sample collection and processing

**Figure 1:**
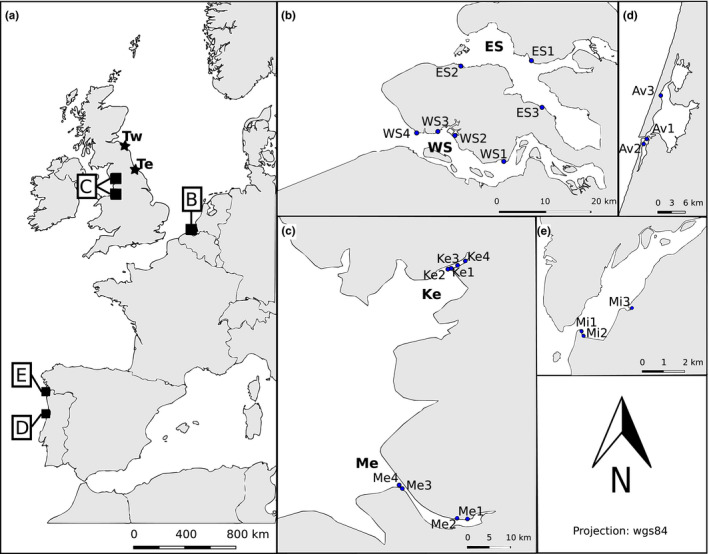
Overview of sample locations, illustrating (a) the overall western European scale, including the location of the Tweed (Tw) and Tees (Te) estuaries, marked by stars (where the study comparing fish detection performance of stomach, sediment, water DNA and traditional netting took place); (b) the Dutch estuaries, Western Scheldt (WS) and Eastern Scheldt (ES); the British estuaries (c), Mersey (Me), Kent (Ke) (Tees and Tweed only present in inset a); the Aveiro (d) and Minho (e) estuaries in Portugal. Small numbered dots within estuaries represent individual collection points for shrimp and sediment samples. Source map: OpenStreetMap [Colour figure can be viewed at wileyonlinelibrary.com]

To evaluate the suitability of trophic contents to assess fish diversity, adult brown shrimp (20–50 mm total length) and sediment samples were collected from the intertidal zone (0–1 m depth) at 21 sites distributed over six estuaries in the Netherlands, Portugal and the United Kingdom (Figure 1), between May and July 2016. Shrimp were collected by push‐net at low tide (±3 hr). Sediment samples were collected with a PVC corer (3.2 mm Ø) from the upper 2‐cm surface layer (which represent the most recent DNA deposits; Limburg & Weider, 2002; Turner, Uy, & Everhart, 2015), and three subsamples were pooled per site to reduce the influence of local heterogeneity (Taberlet, Prud'Homme, et al., 2012). See Siegenthaler et al. (2018) for more details on the sampling protocol. To compare metabarcoding results and seine net surveys, additional shrimp, sediment and water samples (2 L) were collected, in triplicates, from two sites in the Tweed and Tees estuaries in the UK, in May–June 2017 (Figure 1a). In these two sites, seine net surveys were carried out at low tide (±3 hr) to assess fish catch data and for the collection of shrimp for stomach extractions. Surface water samples (0–1 m depth) were collected in sterile 2‐L bottles provided with a 200‐µm nylon mesh. All samples were packed in individual plastic bags and placed on ice for transport and stored at −20°C. Prior to transport, sediment samples were conserved in 96% ethanol.

### 2.2 DNA extraction

In total, 483 stomachs (Supporting Information Table S1) were dissected using flame‐sterilized tools from shrimp that had a visually full stomach. Single shrimp stomachs likely contain only DNA from a limited number of fish due to their small size and fast gut passage time (Feller, 2006; Pihl & Rosenberg, 1984). Up to eight full stomachs were, therefore, pooled prior to DNA extraction (Deagle et al., 2005; Ray et al., 2016), resulting in three extractions per site. Three sites (Av3, Me4 and Ke2) contained only two samples due to a low number of shrimp caught with full stomachs at these locations (see Siegenthaler et al., 2018). Water samples (0.9 L) were filtered using Sterivex filter units (0.22 µm pore size; Merck Millipore) upon arrival to the laboratory (within 4 hr after collection). Pooled stomach (0.25 g) and sediment (10 g) samples were homogenized and DNA extracted using the PowerSoil^®^ DNA isolation Kit (Qiagen) and the PowerMax^®^ DNA Soil Kit (Qiagen), respectively. For the water samples, DNA was extracted from the filters and isolated using the DNeasy PowerWater^®^ DNA isolation Kit (Qiagen). A Qubit fluorometer (Thermo‐Fisher Scientific) was used to assess the DNA concentrations of the purified extracts. DNA extraction and pre‐PCR preparations were performed in separate laboratories from post‐PCR procedures to avoid contaminations.

### 2.3 DNA amplification and high‐throughput sequencing

For the evaluation of *C. crangon* stomach contents to assess fish diversity on a European scale, stomach and sediment sample extracts from the Dutch, Portuguese and UK (except Tweed and Tees), estuaries were amplified using two primer sets: one targeting the mitochondrial cytochrome c. oxidase subunit I (COI) region (henceforth referred to as Leray‐XT; Leray et al., 2013; Wangensteen, Palacín, Guardiola, & Turon, 2018) and another one targeting a hypervariable region in the mitochondrial 12S rRNA gene (henceforth referred to as MiFish; Miya et al., 2015).

The Leray‐XT primer set amplifies a 313‐bp fragment of the COI (Leray et al., 2013) in a broad range of taxa including most metazoan and other eukaryotic groups (Wangensteen et al., 2018). It uses mlCOIintF‐XT (5'‐GGWACWRGWTGRACWITITAYCCYCC‐3') as forward primer (Leray et al., 2013; Wangensteen et al., 2018) and jgHCO2198 (5'‐TAIACYTCIGGRTGICCRAARAAYCA‐3'; Geller, Meyer, Parker, & Hawk, 2013) as reverse primer. The PCR amplification and subsequent HT‐sequencing of this amplicon was conducted as part of a larger project describing the diet of *C. crangon* (Siegenthaler et al., 2018).

The MiFish primer set (Miya et al., 2015) has been developed to target a hypervariable region in the mitochondrial 12S rRNA gene (163–185 bp) and specifically amplifies fish and other vertebrate DNA. For the specific comparison of sample type efficacy, stomach, sediment and water sample extracts from the Tweed and Tees estuaries were amplified using the 12S primer set only, as universal COI primers are known to yield negligible amounts of fish reads when used with extracts from filtered water samples (Grey et al., 2018; Bakker et al., unpublished results).

The PCR mix recipe for the Leray‐XT primer set included 10 µl AmpliTaq gold 360Master mix (Applied Biosystems), 3.2 µg Bovine Serum Albumin (Thermo Scientific), 1 µl of each of the 5 µM forward and reverse tagged primers (including 2–4 leading Ns and 8‐bp sample tags), 5.84 µl H_2_O and 2 µl extracted DNA template (standardized to 5 ng/µl). Twin tagging was used to reduce tag jumping, and different tags were used for sediment and stomach samples from the same site. The PCR profile included an initial denaturing step of 95°C for 10 min, 35 cycles of 94°C for 1 min, 45°C for 1 min and 72°C for 1 min and a final extension step of 72°C for 5 min. PCR products (including two negative controls) with sample tags attached were pooled at equimolar concentration into two multiplex sample pools (sediment sample pool and stomach sample pool) and purified using MinElute columns (Qiagen). Library preparation was performed using the NextFlex PCR‐free library preparation kit (BIOO Scientific), and library quantification was done using the NEBNext qPCR quantification kit (New England Biolabs). Libraries were pooled (along with 0.7% PhiX v3, Illumina, serving as a positive sequencing quality control) in a 1:4 sediment:stomach molar concentration ratio (reflecting the sediment:stomach sample ratio) and sequenced (final molarity of 8 p.m.) on an Illumina MiSeq platform using v2 chemistry (2 × 250 bp paired‐ends).

Prior to the 12S PCR amplification, DNA from the three stomach extractions per site was pooled, resulting in final pools of 16–24 stomachs per sample (with the exception of the Tweed and Tees samples; see Supporting Information Table S1) and standardized to 5 ng/µl. Amplification of the 12S fragment (for the sediment, water and pooled stomach samples) was achieved using a two‐step PCR protocol by first amplifying the amplicon using untagged primers and sequentially amplifying the product of the first PCR with tagged primers to attach a 7‐bp index to each sample (Andruszkiewicz et al., 2017; Miya et al., 2015). Different tags were used for sediment and stomach samples from the same site. Between the two PCR steps, a 5‐time dilution and size selection were performed using MultiScreen® PCR_µ96_ plates (Millipore) to remove any leftover primers. The MiFish primer pair was used for both PCR steps (forward: 5'‐GTCGGTAAAACTCGTGCCAGC‐3'; reverse: 5'‐CATAGTGGGGTATCTAATCCCAGTTTG‐3'; Miya et al., 2015), and the PCR mix recipe was the same as the one mentioned above for the COI amplification. The PCR profile (for both steps) included an initial denaturing step of 95°C for 10 min, 40 cycles of 95°C for 30 s, 60°C for 30 s and 72°C for 30 s and a final extension step of 72°C for 5 min. Two technical PCR replicates were produced per sample, and two negative controls were included. PCR products were pooled into two multiplex sample pools (one pool per PCR replicate), and the pools were sequenced in equimolar concentrations (final molarity of 9 p.m.) along with 0.8% PhiX on Illumina MiSeq platform using v2 chemistry (2 × 150 bp paired‐ends) in accordance with the protocol described above for the COI fragment.

### 2.4 Bioinformatic and data analyses

Bioinformatic analyses were performed using the OBITools metabarcoding software suite (Boyer et al., 2016). Assessment of read quality was done with FastQC, paired‐end read alignment with illuminapairedend, and reads with alignment quality score >40 were retained. Demultiplexing and primer removal was achieved using ngsfilter. Aligned reads with a length of 303–323 bp (for COI) or 140–190 (for 12S) and free of ambiguous bases were selected using obigrep and dereplicated with obiuniq. Chimeras were removed using the uchime‐denovo algorithm (implemented in VSEARCH; Edgar, Haas, Clemente, Quince, & Knight, 2011; Rognes, Flouri, Nichols, Quince, & Mahe, 2016). Amplicon clustering was performed using the SWARM 2.0 algorithm (Mahé, Rognes, Quince, Vargas, & Dunthorn, 2014, 2015 ) with a *d* value of 13 for the COI pipeline, and with a *d* value of 3 for the 12S pipeline. Taxonomic assignment was achieved using the ecotag algorithm (Boyer et al., 2016) on representative sequences for each MOTU to taxa in relation to local reference databases (Wangensteen et al., 2018). The COI database (db COI Sep2017) contained 191,295 filtered COI sequences of eukaryota retrieved from the BOLD database (Ratnasingham & Hebert, 2007) and the EMBL repository (Kulikova et al., 2004). The 12S database (db Miya Sep2017) contained 6,868 sequences from vertebrates retrieved from GenBank. Further refinement of the data was achieved by clustering MOTUs assigned to the same species, the application of abundance renormalization to remove false positives arising from tag switching (Wangensteen & Turon, 2017) and the removal of singletons. Reads not belonging to bony fish taxa (class: Actinopterygii) were removed. To avoid false positives and remove low‐frequency noise, a minimum copy threshold of five reads per sample was applied to the COI data set on a sample‐by‐sample basis (Alberdi, Aizpurua, Gilbert, Bohmann, & Mahon, 2018). False positives were removed from the 12S data set by means of a restrictive approach in which only MOTUs that occurred in both PCR replicates were considered (Alberdi et al., 2018). Remaining reads of the two PCR replicates were merged per sample after filtering. Both the minimum copy threshold of five copies and the restrictive PCR replicate approach can be considered conservative and may have had a negative effect on the detected diversity (Alberdi et al., 2018). Stomach reads were merged per site for the COI data set to obtain comparable data sets between the two markers, based on the same stomachs pools. An overview of the pipelines is reported in Supporting Information Table S2.

Statistical analyses were performed in r version 3.1.3 (https://www.R-project.org/) with the packages vegan (version 2.3‐5; Oksanen et al., 2016) and BiodiversityR (version 2.5‐3; Kindt & Coe, 2005). Multivariate analyses were conducted based on presence–absence data using the PERMANOVA functions adonis and nested.npmanova (Jaccard dissimilarities and 1,000 permutations). MOTU richness was represented as MOTU/species accumulation curves, while differences in the mean number of MOTU detected between samples, countries and estuaries within countries were tested using generalized linear models and Wilcoxon signed rank tests.

## 3 RESULTS

### 3.1 Molecular biodiversity assessment

**Figure 2:**
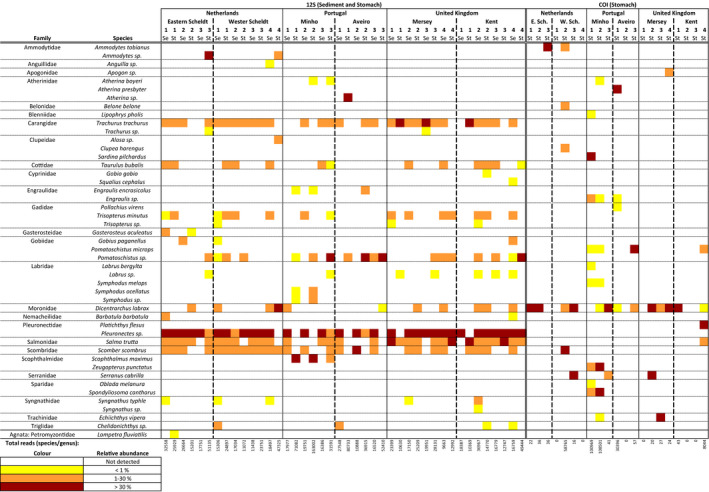
Heatmap of fish species and genera detected in samples taken from Dutch (E. Sch: Eastern Scheldt & W.Sch: Western Scheldt), UK (Mersey & Kent) and Portuguese (Minho & Aveiro) estuaries. The heatmap only shows taxa that are identified at the genus or species level. Fish taxa detected in *Crangon crangon* pooled stomach samples (St) are shown after DNA amplification with two markers: 12S and COI. Sediment samples (Se) are only shown after DNA amplification with the 12S primers since COI amplification resulted in the detection of only 1 MOTU (*D. labrax*) in 1 sample (Minho 1). Colours represent differences in relative read abundances, and the numbers below the columns show the total number of fish reads per sample that could be assigned to the species or genus level [Colour figure can be viewed at wileyonlinelibrary.com]

Total numbers of 2,060,514 and 4,997,391 reads were obtained from 12S and COI amplifications, respectively, after demultiplexing, quality and sequence‐length filtering (reads obtained by HTS: 12S: 9,139,215; COI: 8,895,448). The 12S primers only amplified chordates (Actinopterygii, Agnatha, Aves, Mammalia), while the COI primer pair amplified 40 phyla, including some chordates (Siegenthaler et al., 2018). Percentage of fish (Actinopterygii) reads was high for the 12S primers (75%–89%) and low for the COI primers (sediment: <0.01%, stomach: 7%; Table 1). Taxonomic assignment resulted in a total of 219 Actinopterygii MOTUs identified using the 12S marker, of which 62 were identified to the species or genus level (using the ecotag algorithm; Boyer et al., 2016). Of the 27 Actinopterygii MOTUs detected in the samples using the COI marker, 25 were assigned to the species or genus level. Using the COI marker, only one fish MOTU (*Dicentrarchus labrax*; 15 reads) could be detected in the sediment (and only at one site: Minho 1), and therefore, it was not considered for further analyses. Figure 2 shows a heat map of all MOTUs identified to the species or genus level with the different markers, sample locations and sample media. Large variation is visible in the species detected across sample. For stomach samples, the number of fish species detected was not correlated with the number of stomachs pooled per sample (Spearman correlation; COI: *r* = 0.32, *p* = 0.16; 12S: *r* = 0.15, *p* = 0.52). Notable observations are as follows: the wide occurrence and relatively high abundances of *Pleuronectes* sp., *Salmo trutta*,* Scomber scombrus* and *Trachurus trachurus* in the amplifications with 12S but not with COI; the wide presence of *D. labrax*, which is more effectively detected by the COI marker; and the detection, via 12S, of the river lamprey *Lampetra fluviatilis* in the Eastern Scheldt. The two markers can complement each other in the identification of some taxa to the species level (e.g., the *Atherina* sp. detected via 12S in Aveiro 1 is most likely *A. presbyter*, as inferred by COI). No clear geographical patterns were detected (Supporting Information Figures S1 and S2). Nested PERMANOVA analyses (1,000 permutations) did not show any differences between countries (12S stomach: *F* = 2.28, *p* = 0.08; 12S sediment: *F* = 0.34, *p* = 0.39; COI stomach: *F* = 1.35, *p* = 0.22) and estuaries nested in countries (12S stomach: *F* = 2.50, *p* = 0.54; 12S sediment: *F* = 0.46, *p* = 0.84; COI stomach: *F* = 1.62, *p* = 0.63). COI sediment samples were not tested since fish DNA was only detected in the sediment of one estuary.

**Table 1:** Total number of bony fish (class Actinopterygii), lamprey (class Agnatha) and non‐fish reads detected in samples sequenced using COI and 12S markers. Fish reads are given after quality filtering and removal of false positives

Reads	COI		12S		Tees & Tweed (12S)		
	Sediment	Stomach	Sediment	Stomach	Sediment	Stomach	Water
Bony fish	15	306,997	407,377	799,272	199,254	205,886	119,652
Lamprey	0	0	0	63	28	0	0
Non‐fish	620,310	4,070,069	107,059	95,720	60,574	26,797	38,923
Total	620,325	4,377,066	514,436	895,055	259,828	232,683	158,575
Fish reads (%)	0.002	7.01	79.19	89.31	76.69	88.44	75.46

### 3.2 Comparison of molecular markers and sample types

**Figure 3:**
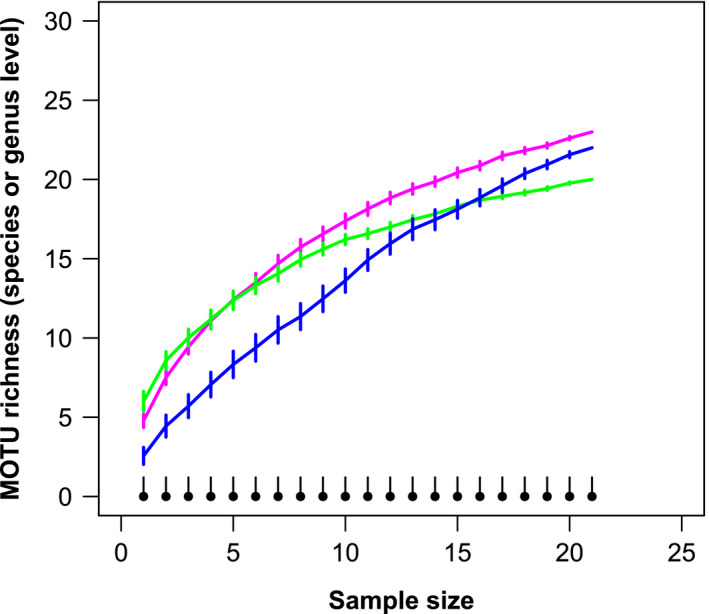
MOTU accumulation curves (±*SE*; 100 permutations) representing the number of bony fish MOTUs identified at the species or genus level detected in sediment and *Crangon crangon* pooled stomach samples analysed with two different markers. Green: Sediment—12S; pink: Stomach—12S; black: Sediment—COI; blue: Stomach—COI. Sediment—COI values are estimated since only 1 MOTU was detected [Colour figure can be viewed at wileyonlinelibrary.com]

**Figure 4:**
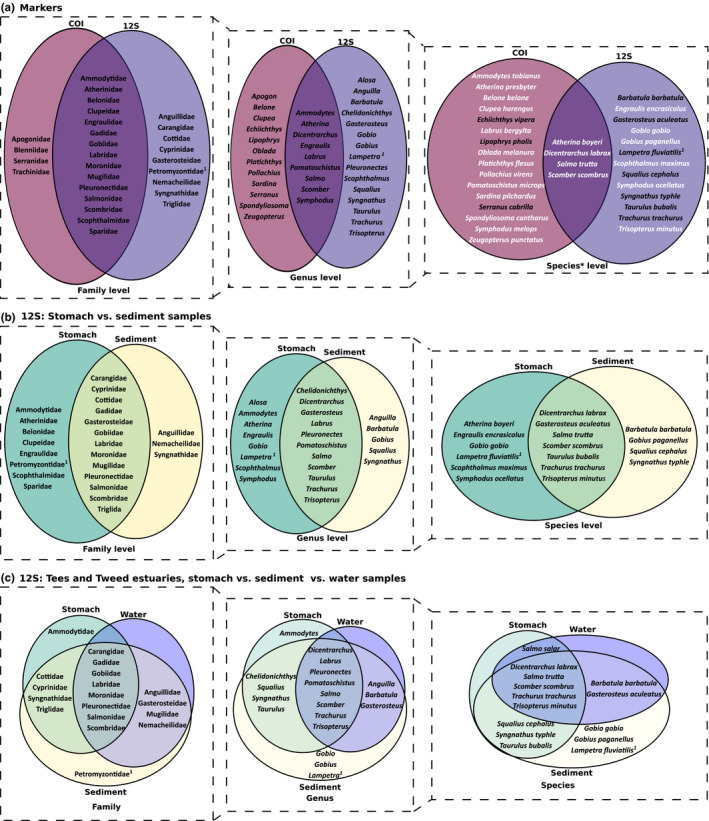
Venn diagrams of fish families, genera and species detected in the (a) DNA of *Crangon crangon* stomach pooled samples amplified with two different markers: 12S and COI; (b) DNA of *C. crangon* pooled stomach and sediment samples amplified with 12S primers; (c) DNA of *C. crangon* pooled stomach, sediment and water samples collected in the Tees and Tweed estuaries in the UK amplified with 12S primers. *Species for which the family was detected with both markers are indicated in white; 1: Class Agnatha [Colour figure can be viewed at wileyonlinelibrary.com]

MOTU accumulation curves showed no differences in the total number of MOTUs identified at the species or genus level between markers (12S/COI) and sample types (sediment/stomach; Figure 3), except for the COI sediment samples (only one MOTU detected). Venn diagrams showed large overlap between markers in the fish families detected, but differences were noted in the species identified (Figure 4a). The total number of fish MOTUs per stomach sample was significantly higher in samples amplified with 12S compared to COI when identified at the family or genus level, but did not differ significantly when only MOTUs identified at the species level were considered (Table 2). Almost half of the fish taxa identified with the 12S marker were observed in both sample types, and this proportion of taxa remained fairly constant among the different taxonomic levels considered (Figure 4b). The total number of taxa identified per 12S‐amplified sample did not differ significantly between sediment and stomach, independently of the taxonomic level of interest (Table 3).

**Table 2:** Differences in mean number of bony fish MOTUs per sample identified at different taxonomic levels in *Crangon crangon* pooled stomach samples amplified with 12S and COI primer pairs. Higher taxonomic ranks include MOTUs identified at the lower levels

	Mean ± *SE* number of MOTU per sample		Wilcoxon signed rank test		
	COI stomach	12S stomach	*N*	V	*p*
All MOTUs	2.2 ± 0.6	6.6 ± 0.6	21	8.0	<0.001*
Family level	2.2 ± 0.6	5.8 ± 0.6	21	13.5	0.001*
Genus level	2.2 ± 0.6	4.9 ± 0.5	21	22.5	0.004*
Species level	2.0 ± 0.5	3.0 ± 0.4	21	76.5	0.177

Asterisks refer to significant comparisons.

**Table 3:** Differences in mean number of bony fish MOTUs per sample amplified with a 12S primer pair and identified at different taxonomic levels between *Crangon crangon* pooled stomach and sediment samples. Higher taxonomic ranks include MOTUs identified at the lower levels. See Supporting Information Table S3 for rarefied data

	Mean ± *SE* number of MOTU per sample		Wilcoxon signed rank test		
	12S stomach	12S sediment	*N*	V	*p*
All MOTUs	6.6 ± 0.6	7.0 ± 0.7	21	82.0	0.61
Family level	5.8 ± 0.6	6.9 ± 0.8	21	68.0	0.17
Genus level	4.9 ± 0.5	5.9 ± 0.7	21	66.0	0.15
Species level	3.0 ± 0.4	4.0 ± 0.5	21	51.5	0.14

More fish species were detected with metabarcoding using 12S than by traditional seine net surveys in the Tees and Tweed estuaries (Table 4). In the Tees estuary, 18 fish taxa (nine of which assigned at the species level) were detected using molecular methods while only five taxa (all identified at the species level) during the seine net survey. Although no MOTUs were detected by metabarcoding that could be assigned to these species, taxa were assigned to the same family or genus level as four of these species (with the exception of *Ammodytes tobianus*). Nine species and four higher taxa (family or genus) were exclusively detected by metabarcoding. In the Tweed estuary, 23 fish taxa (of which 14 assigned to the species level) were detected using molecular methods and only six taxa (all identified at the species level) during the seine net survey. Three species were detected by both methods. Another three species were detected exclusively by netting, but these matched taxa that were assigned to the same family or genus by metabarcoding. Eleven species and six higher taxa (family or genus) were exclusively detected by metabarcoding, including the lamprey *L. fluviatilis*, in the Tweed.

**Table 4:** Fish species detected by 12S metabarcoding and concurrent seine net surveys. Metabarcoding results are based on combined data from sediment, water and *C. crangon* stomach samples amplified with the 12S primer pair (see Figure 4c). Percentage detected shows the percentage of species identified per estuary and, in brackets, the probable percentage of species detected if MOTUs identified to the family or genus level are included

Family	Tees estuary			Tweed estuary	
	Species	Metabarcoding	Netting	Metabarcoding	Netting
Ammodytidae	*Ammodytes tobianus*		√	1	
Anguillidae	*Anguilla* sp.	1		1	
Carangidae	*Trachurus trachurus*	√		√	
Clupeidae	*Clupea harengus*	2	√	2	√
	*Sprattus sprattus*	2	√		
Cottidae	*Taurulus bubalis*	√		√	
Cyprinidae	*Gobio gobio*			√	
	*Squalius cephalus*			√	
Gadidae	Unassigned			2	
	*Trisopterus minutus*	√		√	
Gasterosteidae	*Gasterosteus aculeatus*	√		√	√
Gobiidae	*Gobius paganellus*			√	
	*Pomatoschistus minutus*	1		1	√
Labridae	*Labrus* sp.			1	
Moronidae	*Dicentrarchus labrax*	√		√	
Mugilidae	Unassigned	2		2	
Nemacheilidae	*Barbatula barbatula*	√		√	
Petromyzontidae*	*Lampetra fluviatilis* *			√	
Pleuronectidae	*Platichthys flesus*	2	√	2	√
	*Pleuronectes platessa*	1	√		
Salmonidae	*Salmo salar*			√	√
	*Salmo trutta*	√		√	√
Scombridae	*Scomber scombrus*	√		√	
Syngnathidae	*Syngnathus typhle*	√		√	
Triglidae	*Chelidonichthys* sp.	1		1	
% Detected		50 (94)	28	61 (100)	26

^1^Assigned to genus level. ^2^Assigned to family level. ^*^Class: Agnatha.

Water, sediment and *C. crangon* stomach samples collected from the Tees and Tweed estuaries showed extensive overlap in the species, genera and families detected (Figure 4c). Although some genera were exclusively detected in either sediment (*Gobio*,* Gobius* and *Lampetra*) or stomach (*Ammodytes*) samples, no species, genera or families were solely detected in water samples.

## 4 DISCUSSION

Estuaries are under substantial anthropogenic pressures, including fisheries, pollution, shipping and the spread of invasive species, and thus, the monitoring of their ecological status and variation is essential to safeguard ecosystem functioning and the services provided (Martínez et al., 2007; Sheaves et al., 2015). To improve fish diversity assessment, recent studies have employed molecular tools such as eDNA metabarcoding (Evans & Lamberti, 2017; Thomsen et al., 2012). The present study introduces a novel approach, which benefits from the “natural sampling” properties of a generalist predator/scavenger and the power and speed provided by metabarcoding.

Results show that metabarcoding of environmental and trophic samples was much more effective in determining the local fish community structure than traditional seine net surveys, in line with a growing body of work on the use of eDNA in fish surveys (e.g., Boussarie et al., 2018; Thomsen et al., 2012; Valentini et al., 2016). Twice the number of species and more than three times the number of taxa (assigned to the genus of family level) were detected using 12S‐metabarcoding on a combination of sediment, shrimp stomach and water samples compared to concordant seine net surveys. Furthermore, fish taxa detected by metabarcoding, but not by traditional netting, included some important taxa for conservation such as *Anguilla* and *Lampetra*. Although it was not possible to identify all species caught during the seine net surveys at the species level using molecular assignment only, the family/genus‐level identification indicates that the DNA of these species was indeed amplified, hence allowing in most cases indirect inference on species presence. Improvements of the reference database or marker's taxonomic resolution will be required to attain unambiguous, direct molecular identification of these taxa at the species level (Alberdi et al., 2018; Shaw et al., 2016).

The fish diversity detected during this study, by using a combination of sediment and *C. crangon* stomach samples, reflects a typical European estuarine community, including estuarine residents (e.g., *A. tobianus* and *Pomatoschistus microps*) and species that use estuaries as a nurseries and/or feeding grounds (e.g., *D. labrax* and *Pleuronectes platessa*), migrate through them (e.g., *Anguilla* sp. and *Salmo trutta*) or behave as marine or freshwater stragglers (e.g., *Scomber scombrus*,* Trachurus trachurus* and *Gobio gobio*; Elliott & Dewailly, 1995; Elliott et al., 2007; Maes, Stevens, & Ollevier, 2005). Several species detected, such as eel (*Anguilla* sp.), European plaice (*P. platessa*), sea bass (*D. labrax*) and Atlantic mackerel (*S. scombrus*), are important commercial targets. Considering the small size of *C. crangon* caught (20–50 mm total length; Siegenthaler et al., 2018), it is surprising to see that such a small shrimp feed on a large range of fish species including several known and potential own predators (e.g., *P. microps and D. labrax*; Cattrijsse, Dankwa, & Mees, 1997). Consumption of fish tissue is, therefore, likely a combination of scavenging on adults and direct predation on juveniles/larvae (Ansell, Comely, & Robb, 1999; Siegenthaler et al., 2018; van der Veer & Bergman, 1987). Although soft bottom habitats were sampled, several hard‐bottom‐associated species were detected (e.g., *Lipophrys pholis* and *Labrus bergylta*) which could have been occasional visitors from nearby rocky shores (e.g., rocky outcrops located near the mouth of the Minho estuary) or were captured/scavenged by shrimp migrating in and out the estuaries (Al‐Adhub & Naylor, 1975). In addition to DNA originated from the biota present in the estuaries, DNA detected in the sediment and water samples might also have been transported *post‐mortem* from other adjacent areas by river run‐off or during tidal movements (Barnes & Turner, 2016).

Besides site‐specific variation, no geographical patterns were detected during this study. The focus of the present study is the assessment of a trophic metabarcoding approach to evaluate fish diversity and does not aim to provide a detailed geographical comparison, for which a higher sample density would be required. Also, European estuaries show high similarities in fish assemblages, especially for common/abundant species (Elliott & Dewailly, 1995). Differences in rare species may also have been masked by pooling of the samples, though pooling is still considered suitable for among‐site comparison of representative fish communities (Sato, Sogo, Doi, & Yamanaka, 2017). Taking these considerations into account, further studies with expanded geographical range and seasonal samplings are likely to yield the full breadth of European estuarine ichthyofaunal complexity.

### 4.1 Marker and DNA medium choice

The results of this study add to the growing body of evidence underpinning the efficacy of molecular tools to effectively detect biodiversity (Andruszkiewicz et al., 2017; Evans & Lamberti, 2017; Taberlet, Coissac, et al., 2012), but also show that a combination of multiple markers and different sample types are required to gain a comprehensive understanding of the study system (Alberdi et al., 2018; Deagle, Jarman, Coissac, Pompanon, & Taberlet, 2014; Shaw et al., 2016). Environmental DNA is generally only present in trace amounts in the environment and the concentration of DNA can differ between media, influencing the detectability of taxa (Shaw et al., 2016; Taberlet, Prud'Homme, et al., 2012; Turner et al., 2015). No major differences were detected in the number of fish species identified with the 12S primer pair between the sediment samples and *C. crangon* stomach contents. In the samples from the Tees and Tweed estuaries, more fish species were detected in the eDNA extracted from the sediment than from the water and no species, genera or families were exclusively detected in the water samples. Differences in fish detectability between these sample types could be due to the generally higher concentration and temporal persistence of DNA in sediment samples compared to the water column (Turner et al., 2015) or caused by differences in the volumes of substrates used (Shaw et al., 2016). It should be noted, however, that only a limited number of water samples was taken due to the reliance of this study on previously collected samples (Siegenthaler et al., 2018). Since water sampling has been the main technique for eDNA‐based fish surveys (e.g., Shaw et al., 2016; Valentini et al., 2016), further research comparing eDNA extracted from trophic and water samples might be required for a more in‐depth comparison.

The differences in species detection between the 12S and COI markers are likely due to a combination of primer bias, differences in reference database completeness and the taxonomic resolution of the markers (Alberdi et al., 2018; Taberlet, Coissac, et al., 2012). The COI data were collected as part of a larger study on the diet of the brown shrimp (Siegenthaler et al., 2018), while the 12S data were specifically collected for the detection of fish DNA in shrimp stomach samples. Furthermore, due to slight variations in laboratory procedures (e.g., the requirement of two‐step PCR protocol for the 12S primer), two different strategies were used to compensate for PCR‐sequencing errors, contaminants and false positives: a high copy number threshold (>5 reads on a sample‐by‐sample basis) for the COI marker and a restrictive PCR replicate strategy for the 12S marker (Alberdi et al., 2018). These differences could have had an influence on the differences in species richness, due to chimaeras, PCR stochasticity and contaminations, detected between the two different markers. Comparisons between the two markers should, therefore, be considered with caution. Yet, taking this into consideration, it can be argued that of the markers used during this study, COI was more accurate for species‐level detection than 12S, due to its better taxonomic resolution, which ensures high discrimination power at the species level, and the availability of an exhaustive and well‐curated reference database (BOLD; Ratnasingham & Hebert, 2007). Its use was, however, severely limited in samples where the relative amount of fish DNA was low (e.g., sediment samples) due to the nearly universal taxonomic breadth of the COI primers used (Wangensteen et al., 2018). Interestingly, more teleost DNA was amplified using COI primers from the stomachs samples than from the sediment samples, even though no blocking primers were used to block *C. crangon* DNA (Ray et al., 2016). The sediment samples were, however, dominated by reads of a wide range of protist and invertebrate meiofaunal taxa (Siegenthaler et al., 2018). While the Leray‐XT primer pair (COI) has been developed for universality (Wangensteen et al., 2018), the MiFish primer pair (12S) has been developed to specifically detect eDNA from fishes (Miya et al., 2015). The main limitations of 12S‐based markers are, however, that 12S rDNA teleost coverage is relatively poor in the NCBI nucleotide database (Andruszkiewicz et al., 2017) and the fact that 12S‐based markers have lower taxonomic resolution for fish species than other markers (Pesole, Gissi, Chirico, & Saccone, 1999; Shaw et al., 2016). An appropriate choice of marker and sample medium are, therefore, intertwined and depend on the research question (e.g., rarity of the taxa of interest), the taxonomic resolution required and the availability of resources to improve reference databases.

### 4.2 Applications in fisheries and environmental sciences

Fisheries science requires tools that provide reproducible data on species diversity, stock size and demographic information of the area under study, preferably for minimal cost and labour. Traditional methods are not always able to provide this, as results vary highly with the sampling technique used, including type of gear and depth of fishing. They are, furthermore, often expensive and labour intensive (Cotter et al., 2004; Courrat et al., 2009; Lapointe et al., 2011; Thomsen et al., 2012). Presently, eDNA metabarcoding techniques also show limitations for several of these requirements since they are not able to assess population structure and fish condition, quantitative data on biomass or number of individuals, nor they provide real‐time and fine‐scale information (Evans & Lamberti, 2017; Shaw et al., 2016). Standardization of the molecular and analytical methods used for metabarcoding is also required for reproducibility and to allow for combination of data from different studies (Gilbert, Jansson, & Knight, 2014; Lear et al., 2018; Leese et al., 2016). On the positive side, eDNA samples are easier to collect, require lower sampling effort and are less labour intensive than traditional fishing methods (Boyer et al., 2015; Evans, Shirey, Wieringa, Mahon, & Lamberti, 2017; Smart et al., 2016). In addition, molecular monitoring of fish populations (either from environmental or stomach samples) does not require taxonomic expertise, is more objective than traditional methods and, in general, results in more species detected than conventional methods (Boussarie et al., 2018; Thomsen et al., 2012; Valentini et al., 2016; this study). Besides species distribution assessments, the use of eDNA is especially useful for the detection and monitoring of rare (e.g., *Anguilla* and *Agnatha*) and invasive species (Evans & Lamberti, 2017), as required for compliance with environmental policy, such as the EU Habitat Directive. Since the costs of molecular consumables continue to decline and the speed of sequencing analyses and bioinformatic pipelines increases, molecular techniques (either based on environmental or stomach samples) have the potential to become a valuable complement to traditional sampling methods (Evans et al., 2017; Smart et al., 2016).

One of the appealing aspects of using a variety of media for environmental and community DNA collection is that, while these can easily be collected simultaneously, they can reveal different levels of information about the community under investigation. Environmental DNA extracted from water samples usually integrates information over large spatial scales but has a low temporal resolution due to the high dispersion and low persistence of DNA in sea water (Barnes & Turner, 2016; Thomsen et al., 2012). Sediment samples, on the other hand, can store and conserve DNA for months to years and their high spatial heterogeneity, as often detected between samples, might provide information on small spatial scales (Taberlet, Prud'Homme, et al., 2012; Turner et al., 2015). Nevertheless, estuaries act as river catchment areas and sediments store organic matter from upstream freshwater habitats. Resuspension and horizontal transport of eDNA could thus influence interferences made from both sediment and aqueous eDNA (Barnes & Turner, 2016; Turner et al., 2015), especially in dynamic systems such as estuaries.

This study is the first to bring into the scene another promising medium: the gut contents of generalist predators or scavengers. In the case of *C. crangon,* DNA extracted from its stomach contents will likely provide recent information since shrimp have a relatively fast gut passage time (4–20 hr; Feller, 2006; Pihl & Rosenberg, 1984; van der Veer & Bergman, 1987) and digested DNA degrades rapidly (Deagle, Eveson, & Jarman, 2006; Moran, Orth, Schmitt, Hallerman, & Aguilar, 2016). The area “sampled” by a pool of *C. crangon* will likely provide information on a larger spatial scale than acquired by a sediment sample since the shrimp actively moves around during night‐time and shows tidal and seasonal migrations (Al‐Adhub & Naylor, 1975; Donk & Wilde, 1981; Henderson & Holmes, 1987). Furthermore, the effect of resuspension and horizontal transport of upstream eDNA is less influential than in other DNA media since, as a scavenger, *C. crangon* mainly consumes solid tissues which should show a lower dispersion than extracellular eDNA. Compared to DNA extracted from other environmental sources, community DNA extracted from guts will mainly represent the live community present in the system (or recently deceased in the case of scavenging) instead of the mix of cellular and extracellular DNA from different origins, which generally constitute eDNA (Barnes & Turner, 2016). Finally, the fact that the “sampled biodiversity” is naturally encapsulated in the guts of a shrimp, from field sampling all the way to DNA extraction in the laboratory, represents a significant way to streamline and by‐pass many of the fastidious steps required to reduce degradation and contamination when sampling water; a fact that is often underemphasized in eDNA research.

The application of gut metabarcoding has the potential of becoming a powerful tool in biodiversity assessment applications, such as in the case of bioindicators and commercially important taxa (e.g., teleosts). Shrimp can be collected in large numbers without extra effort or costs as bycatch during existing fish surveys (Gamito & Cabral, 2003). Also, the application of fish‐specific markers (e.g., MiFish; Miya et al., 2015) or blocking primers (Vestheim & Jarman, 2008) can greatly reduce the labour required (stomachs do not need to be dissected with precision since shrimp DNA will not be amplified), making the costs of trophic eDNA comparable to other eDNA media. Pooling individual stomachs prior to DNA extraction might also reduce the cost and effort required. Nevertheless, this approach should be most advisable when using primers that are more specific to the taxon of interest (such as the 12S primers used here), in order to avoid the risk of missing the detection of rare prey species (Sato et al., 2017).

The initial results of this and other studies, using molecular or traditional techniques (Boucek & Rehage, 2014; Lasley‐Rasher et al., 2015; Schnell et al., 2012), show that diet‐based fish diversity techniques are very promising. Certainly, their application remains dependent on the availability of a suitable, ubiquitous and accessible, “sampling species,” which requires expanded knowledge of ecological interactions, such as predator–prey dynamics, secondary predation, and the ecology and physiology of both the predator and prey species to assess predator/prey‐related biases (Calvignac‐Spencer, Leendertz, Gilbert, & Schubert, 2013; Schnell et al., 2015; Siegenthaler et al., 2018). Nevertheless, it is clear that eDNA can be used as a bio‐assessment tool for fisheries sciences to complement traditional sampling schemes, to improve species distribution assessment and to monitor invasive and rare species, at competitive costs. The implementation of an approach that interweaves high‐throughput metabarcoding with the “natural sampling capacity” derived from feeding activities of opportunistic/scavenging species may in the near future offer the right blend of power, speed and cost‐effectiveness for large‐scale, routine applications.

## AUTHOR CONTRIBUTIONS

A.S. participated in the study design; carried out the field, laboratory and molecular work, data and statistical analyses; and wrote the manuscript; O.S.W. participated in the molecular work, designed the bioinformatics pipeline and assisted in the statistical analyses; A.Z.S participated in the sampling and molecular work; C.B. participated in the study design; L.C. coordinated the net surveys. S.M. conceived and designed the study and contributed to writing. All authors provided critical comments on the manuscript and gave final approval for publication.

## Data Availability

The data set, including sequences, taxonomic assignment and abundances for all MOTUs in every sample, has been deposited in Dryad (https://doi.org/10.5061/dryad.7498b88). Custom R scripts are publicly available from https://github.com/Andjin/Crangon-diet-analysis and https://github.com/metabarpark for scripts related to the bioinformatics pipeline.
