# Supplementary material for: Metabarcoding of shrimp stomach content: Harnessing a natural sampler for fish biodiversity monitoring
Source: Mol Ecol Resour. 2018 Nov 9;19(1):206–20. doi: 10.1111/1755-0998.12956 (PMC7379652; doi:10.1111/1755-0998.12956)
Supplement: Supplementary file 1 [file MEN-19-206-s001.pdf]

MOLECULAR ECOLOGY RESOURCES

Supplemental Information for:

Metabarcoding of shrimp stomach content: harnessing a natural sampling device for fish biodiversity monitoring

Andjin Siegenthaler, Owen S. Wangensteen, Ana Z. Soto, Chiara Benvenuto, Laura Corrigan2 & Stefano Mariani

Table of Contents:

Supplementary table S1	Page 2
Supplementary table S2	Page 3
Supplementary figure S1	Page 4
Supplementary figure S2	Page 5
Supplementary table S3	Page 6

MOLECULAR ECOLOGY RESOURCES

Supplementary table S1. Number of *C. crangon* stomachs pooled per sample. Each sample (except Tees and Tweed samples) consist of a combination of 3 extractions of up to 8 stomachs.

	Estuary	Sample	Site	N stomachs
Netherlands	Eastern Scheldt	1	ES1	24
		2	ES2	21
		3	ES3	24
	Western Scheldt	1	WS1	24
		2	WS2	20
		3	WS3	17
		4	WS4	20
Portugal	Minho	1	Mi1	24
		2	Mi2	24
		3	Mi3	24
	Aveiro	1	Av1	20
		2	Av2	23
		3	Av3	16
United Kingdom	Mersey	1	Me1	16
		2	Me2	24
		3	Me3	24
		4	Me4	24
	Kent	1	Ke1	21
		2	Ke2	16
		3	Ke3	24
		4	Ke4	23
	Tees†	1	Te1	5
		2	Te1	5
	Tweed†	1	Tw1	8
		2	Tw1	8
		3	Tw1	4

† Multiple biological replicates were taken from one site

MOLECULAR ECOLOGY RESOURCES

Supplementary table S2. Summary of the bioinformatic pipelines used for both markers. owi_recount_swarm and owi_add_taxonomy are custom R scripts available at http://github.com/metabarpark.

	PIPELINE FOR COI	PIPELINE FOR 12S
PCR amplification	Leray-XT primers. Single PCR protocol. Three amplifications per site (6- 8 stomachs)	MiFish primers. 2-PCR protocol. 2 replicates per sample. One amplification per site (16-24 stomachs)
Library preparation	NEXTflex (BIOO). Separate libraries for sediments and stomach samples.	NEXTflex (BIOO). 2 replicate libraries.
HT Sequencing	Illumina MiSeq 2x250 bp	Illumina MiSeq 2x150 bp
Raw sequences QC	fasttqc<br>fastx_trimmer	fasttqc<br>No trimming needed
PE alignment	illuminapairedend	illuminapairedend
Demultiplexing	obiannotate/obisplit<br>ngsfilter	obiannotate/obisplit<br>ngsfilter
Length filter	obigrep 300-320 bp	obigrep 140-190 bp
Dereplication	obiuniq	obiuniq
Rename identifiers	obiannotate	obiannotate
Chimera removal	vsearch uchime_denovo	vsearch uchime_denovo
Clustering	SWARM v2 d=13<br>obitab<br>owi_recount_swarm<br>delete singletons	SWARM v2 d=3<br>obitab<br>owi_recount_swarm<br>delete singletons
Taxonomic assignment	ecotag using db COI Sep2017	ecotag using db Miya Sep2017
Add higher taxa	owi_add_taxonomy	owi_add_taxonomy
Final refinements	Blank correction<br>Removal of non-fish MOTUs<br>Abundance renormalization<br>Collapse multi-MOTU species<br>Minimal abundance filtering (>4 reads)<br>Collapse biological replicates per site by adding abundances	Blank correction<br>Removal of non-fish MOTUs<br>Abundance renormalization<br>Removal of MOTUs detected in just one of the replicates (>1 read per sample)<br>Collapse replicates by adding abundances

MOLECULAR ECOLOGY RESOURCES

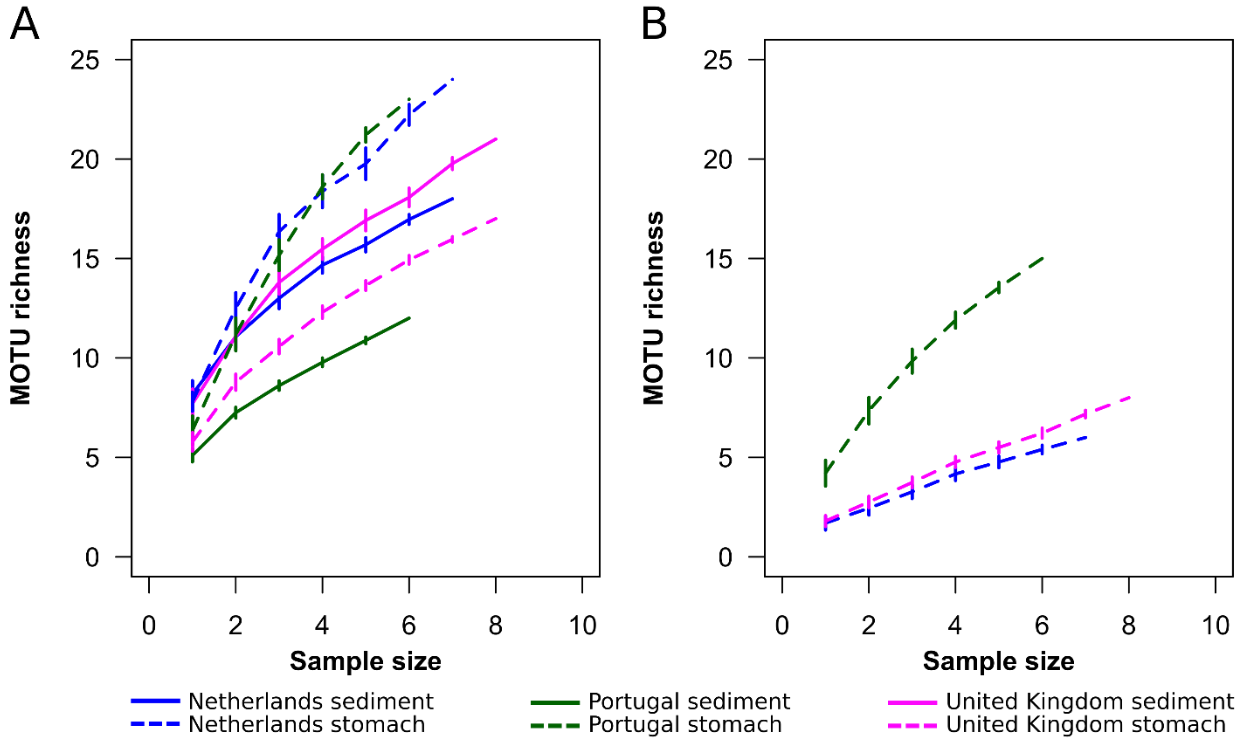

Supplementary figure 1. MOTU accumulation curves (±SEM; 100 permutations) for the number of bony fish MOTUs in DNA extracted from sediment and *Crangon crangon* pooled stomach samples collected in different countries. Samples were analyzed with two different primer pairs: 12S (A); COI (B). Only one fish was detected in COI sediment samples.

MOLECULAR ECOLOGY RESOURCES

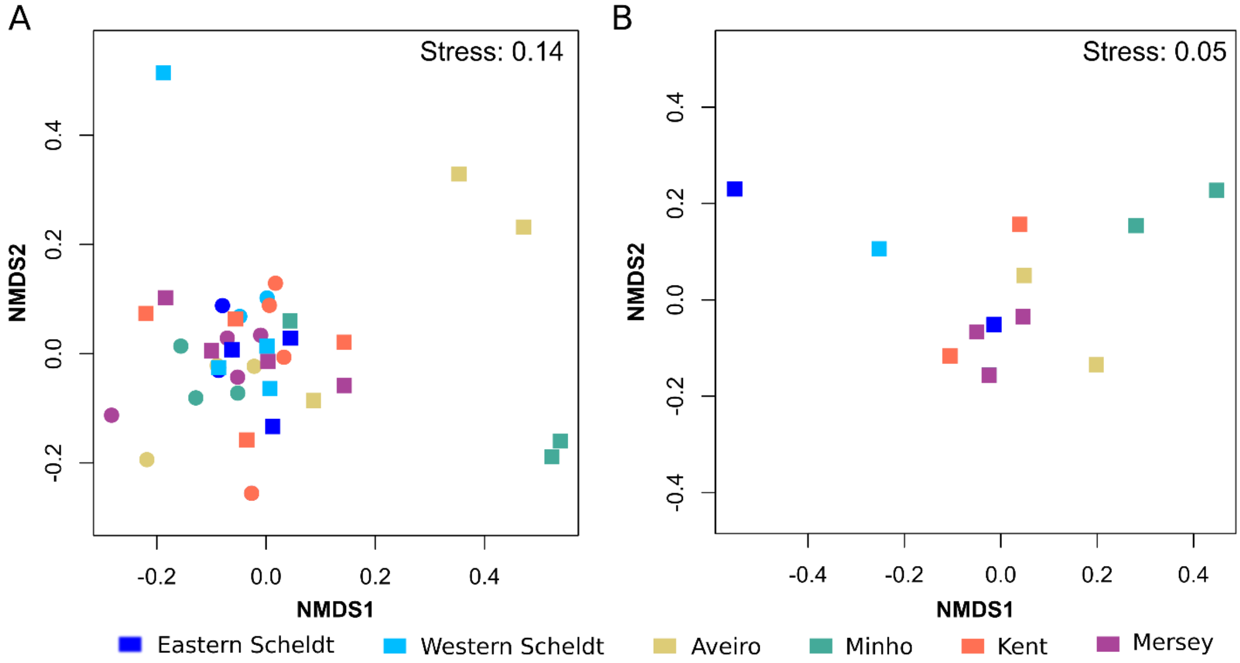

Supplementary figure 2. Multidimensional scaling analysis (based on Jaccard dissimilarities) of bony fish MOTUs detected in sediment (dots) and *Crangon crangon* pooled stomach samples (squares), after DNA amplification with (A) 12S (B) COI primer pairs. Estuaries are identified by colour.

MOLECULAR ECOLOGY RESOURCES

Table S3. Differences in rarefied mean number of bony fish MOTUs per sample, amplified with a 12S primer pair, and identified to different taxonomic levels between *Crangon crangon* pooled stomach and sediment samples. Higher taxonomic ranks include MOTUs identified at the lower levels. Unassigned reads are not included.

	**Mean ± SE number of MOTU per sample**		**Rarefaction**	**Wilcoxon signed rank test**		
	**12S stomach**	**12S sediment**	**N reads**	**N**	**V**	**P**
All MOTUs	6.5 ± 0.6	7.0 ± 0.7	9600	21	127.0	0.42
Family level	5.5 ± 0.5	6.0 ± 0.6	9600	21	122.0	0.29
Genus level	4.7 ± 0.5	5.6 ±0.7	6700	21	127.0	0.20
Species level	3.0 ± 0.4	4.0 ± 0.5	2810	21	146.5	0.13
